# Self‐Leadership Based on Caring in Primary Nursing: Developing and Validating a Conceptual Model Through a Delphi Study

**DOI:** 10.1155/jonm/7740914

**Published:** 2026-08-12

**Authors:** Mahmud Ady Yuwanto, Iyus Yosep, Iqbal Pramukti, Ati Surya Mediawati

**Affiliations:** ^1^ Doctoral Study Program in Medical Science, Faculty of Medicine, Universitas Padjadjaran, Sumedang, Indonesia, unpad.ac.id; ^2^ Department of Fundamental Nursing, Faculty of Health Sciences, Universitas Dr. Soebandi, Jember, Indonesia; ^3^ Department of Community and Mental Health Nursing, Faculty of Nursing, Universitas Padjadjaran, Sumedang, Indonesia, unpad.ac.id; ^4^ Department of Fundamental Nursing, Faculty of Nursing, Universitas Padjadjaran, Sumedang, Indonesia, unpad.ac.id

**Keywords:** caring, Delphi study, nursing leadership, primary nursing, professional agency, self-leadership

## Abstract

**Aim:**

To develop and validate a conceptual model of self‐leadership based on caring for primary nursing practice by integrating empirical findings with self‐leadership (Manz–Neck) and caring (Watson–Swanson) theories.

**Design:**

A preliminary model was developed through the integration of empirical findings from previous qualitative and quantitative studies and informed by self‐leadership theory and caring theories.

**Methods:**

A preliminary model was developed by synthesising empirical findings from previous qualitative and quantitative studies with self‐leadership (Manz–Neck) and caring (Watson–Swanson) theories to generate an initial conceptual framework, which was subsequently evaluated by an expert panel comprising nursing academics, nurse managers, senior clinical nurses, and primary nurses.

**Results:**

The Delphi process refined the model by positioning caring enactment as the central mechanism through which self‐leadership capacities are translated into accountable professional agency and practice outcomes, while reflexive feedback supports continuous leadership development through reflective learning and practice experience.

**Conclusion:**

The model advances nursing leadership scholarship by extending self‐leadership theory beyond its traditional intrapersonal focus and conceptualising caring enactment as the mechanism through which leadership capacities become accountable professional practice. It provides a theoretically grounded framework for understanding how frontline nurses translate self‐leadership capacities into accountable professional action and sustained practice outcomes.

**Implications for Nursing Management:**

The model offers nursing managers a practical framework for strengthening frontline leadership through the development of self‐leadership capacities, caring enactment, and accountable professional agency. Its application may inform leadership development programmes, reflective supervision, mentorship initiatives, and organisational resources such as leadership support, protected time for reflective practice, and role clarity to strengthen primary nursing implementation aimed at improving continuity of care, quality of nursing care, and workforce sustainability.

## 1. Introduction

Contemporary healthcare systems are increasingly challenged by rising patient acuity, workforce shortages, growing care complexity, and escalating expectations for safe, coordinated, and person‐centred care [1]. Within these demanding environments, nurses are expected not only to provide high‐quality clinical care but also to demonstrate leadership through care coordination, clinical decision‐making, patient advocacy, and professional accountability [2, 3]. Consequently, leadership is increasingly recognised as a core component of professional nursing practice rather than a responsibility confined to formal managerial roles [4, 5].

Among contemporary leadership approaches, self‐leadership has received growing attention as a means of strengthening nurses’ capacity for self‐regulation, self‐motivation, professional responsibility, and adaptive performance. Evidence suggests that self‐leadership contributes to work engagement, resilience, innovation, professional competence, and job satisfaction among nurses [6, 7]. However, existing scholarship has primarily conceptualised self‐leadership as an intrapersonal capability, offering limited explanation of how self‐leadership capacities are translated into observable professional actions within everyday nursing practice [8, 9]. Although self‐leadership strengthens nurses’ capacity for self‐regulation, intrinsic motivation, and autonomous decision‐making, these capabilities do not automatically translate into professional leadership behaviours. Their enactment depends on mechanisms that connect intrapersonal capacities with relational nursing practice, an area that remains insufficiently explained in existing nursing leadership literature.

Caring remains the philosophical and disciplinary foundation of nursing and is central to how nurses establish therapeutic relationships, respond to patient needs, and deliver person‐centred care. Caring theories proposed by Watson and Swanson describe caring as a relational and ethical process through which nurses support the wellbeing of patients and families [10–13]. Contemporary evidence further demonstrates that caring contributes to positive patient experiences, trust, quality of care, and professional fulfilment among nurses [14, 15]. Despite its recognised importance, caring is most often discussed as a professional value, ethical commitment, or desired outcome of practice rather than as an active mechanism through which leadership is enacted [16, 17]. Theoretically, caring extends beyond an ethical value by enabling professional leadership in everyday nursing practice. However, existing caring theories explain why nurses care rather than how caring operationalises self‐leadership.

This separation represents an important conceptual gap in nursing leadership because the mechanism through which self‐leadership is enacted through caring remains insufficiently understood [9, 18]. Self‐leadership theories explain how nurses regulate their own thoughts, behaviours, and motivations, whereas caring theories explain how nurses engage with patients through therapeutic and relational practice [19]. However, the mechanism through which self‐leadership capacities are translated into accountable professional actions through caring remains insufficiently explained in existing nursing leadership scholarship [20, 21]. As a result, existing frameworks provide only partial explanations of how leadership becomes visible in nursing practice beyond individual attributes, competencies, or formal leadership positions [22, 23].

The gap is particularly relevant within primary nursing systems, where nurses assume responsibility for coordinating care, maintaining continuity of care, exercising clinical judgement, and developing sustained therapeutic relationships with patients [24, 25]. These responsibilities require nurses to integrate leadership, accountability, and caring within everyday clinical practice [26, 27]. Nevertheless, existing nursing leadership models have not adequately explained how contextual conditions support self‐leadership, how caring operationalises these capacities into accountable professional actions, and how reflective learning sustains leadership development within a coherent conceptual framework for primary nursing [9, 18]. In primary nursing, caring serves as the relational mechanism through which self‐leadership is enacted as accountable professional practice and strengthened through reflective learning.

Addressing this limitation is important for both nursing theory and nursing management. A clearer understanding of how self‐leadership is enacted through caring may provide new insights into leadership development, professional accountability, workforce sustainability, and quality improvement initiatives [28]. Building on previous empirical findings and theoretical perspectives, this study proposes that caring functions as the central mechanism through which self‐leadership capacities are translated into professional agency and practice outcomes. By positioning caring as an enactment process rather than solely a professional value or outcome, the proposed framework extends current self‐leadership literature and offers a nursing‐specific explanation of frontline leadership within primary nursing practice.

To address this theoretical and practical gap, this study developed and conceptually validated an integrated self‐leadership‐based caring model for primary nursing practice. The preliminary model was developed by synthesising empirical findings from preceding qualitative and quantitative research phases with self‐leadership (Manz–Neck) and caring (Watson–Swanson) theories and was subsequently refined and validated through a two‐round Delphi process. The study aimed to establish a theoretically grounded conceptual framework that explains how self‐leadership is enacted through caring to strengthen accountable professional practice in primary nursing.

## 2. Methods

### 2.1. Study Design

This study employed a two‐round Delphi design to develop and conceptually validate a self‐leadership‐based caring model for primary nursing practice. The Delphi technique was selected because it enables the systematic evaluation and refinement of conceptual models through iterative consultation with experts, facilitating consensus on conceptual relevance, clarity, coherence, and theoretical consistency. Its application is well established for developing and validating conceptual frameworks in health research [29, 30]. Consistent with the purpose of conceptual model development, the study focused on achieving expert consensus regarding the structure, content validity, and theoretical consistency of the proposed framework rather than testing causal relationships or intervention effectiveness. Consensus criteria were established a priori using predefined thresholds for content validity and agreement to ensure methodological transparency and consistency throughout the Delphi process.

### 2.2. Model Development

The preliminary model was developed through the integration of empirical and theoretical evidence generated from a broader programme of research on self‐leadership and caring in primary nursing practice. Empirical inputs were derived from previously completed qualitative and quantitative studies exploring self‐leadership among primary nurses and were synthesised to identify recurring concepts, relationships, and theoretical patterns relevant to primary nursing practice. These findings were subsequently synthesised with concepts derived from the self‐leadership theory proposed by Manz and Neck and the caring theories developed by Swanson [10, 12, 31, 32].

The integration process resulted in a preliminary framework comprising five interrelated domains: contextual enablers, core self‐leadership capacities, caring enactment, primary nurse professional agency, and practice outcomes supported by a reflexive feedback process. During this synthesis, caring enactment emerged as the central conceptual mechanism linking core self‐leadership capacities with accountable professional practice, while reflexive feedback was incorporated to represent continuous professional learning. To facilitate Delphi evaluation, the preliminary framework was operationalised into 20 conceptual statements representing the definitions, functions, and relationships among model domains.

Before Delphi validation, the preliminary model underwent internal conceptual review by the research team to ensure theoretical coherence, logical consistency, and alignment between empirical findings and the proposed conceptual structure. The refined preliminary model subsequently served as the basis for expert evaluation during the Delphi process.

### 2.3. Delphi Panel

Experts were recruited using purposive sampling through professional networks and institutional recommendations to ensure representation from complementary areas of expertise relevant to the proposed model. Eligibility criteria included a minimum of 10 years of professional experience and recognised expertise in nursing leadership, caring science, nursing management, primary nursing practice, or professional nursing development through academic, managerial, or advanced clinical roles. Eight experts participated in the Delphi process, a panel size considered appropriate for conceptual model validation because Delphi studies emphasise the quality and diversity of expert judgement rather than statistical representation [30, 33].

Experts were purposively selected to maximise conceptual diversity rather than numerical representation. The panel included nursing academics, nurse managers, senior clinical nurses, and primary nurses from different healthcare and academic institutions in Indonesia, ensuring variation in professional expertise and clinical perspectives relevant to primary nursing leadership. The panel size was considered appropriate because Delphi studies focussing on conceptual model validation commonly involve small panels of highly qualified experts when the objective is to achieve depth of judgement rather than statistical representation.

### 2.4. Delphi Procedures

#### 2.4.1. Round 1

During Round 1, experts received the preliminary conceptual model and a structured evaluation form containing 20 conceptual statements organised across five model domains. Experts were asked to assess the relevance and conceptual clarity of each statement and to provide written comments regarding definitions, relationships among components, conceptual omissions, and potential areas for refinement. They also evaluated whether the proposed relationships among model domains were conceptually logical, theoretically coherent, and appropriate for explaining leadership enactment in primary nursing practice. The structure of the Delphi evaluation instrument and assessment criteria is presented in Table 1.

**TABLE 1:** Characteristics of Delphi expert panel (*N* = 8).

Expert code	Professional role	Highest qualification	Area of expertise	Years of experience
E1	Nursing academic	Phd	Nursing leadership	18
E2	Nursing academic	Phd	Caring science	16
E3	Nursing manager	Master’s degree	Nursing management	20
E4	Senior clinical nurse	Master’s degree	Clinical leadership	17
E5	Senior clinical nurse	Bachelor’s degree	Clinical nursing practice	15
E6	Primary nurse	Bachelor’s degree	Primary nursing practice	12
E7	Primary nurse	Bachelor’s degree	Primary nursing practice	11
E8	Primary nurse	Bachelor’s degree	Primary nursing practice	13

#### 2.4.2. Round 2

Following analysis of Round 1 findings, a revised version of the model was prepared incorporating expert recommendations. The revised framework, together with a summary of Round 1 findings and the corresponding conceptual revisions, was redistributed to all panel members for re‐evaluation. Experts were invited to review the revised model and indicate their level of agreement regarding the conceptual structure, coherence, relevance, and interrelationships among model domains. The purpose of Round 2 was to confirm consensus, evaluate the stability of the revised framework, and determine whether the conceptual revisions adequately addressed expert feedback from Round 1.

### 2.5. Data Analysis

Experts rated each statement using a four‐point relevance scale ranging from 1 (not relevant) to 4 (highly relevant). A four‐point scale was selected to minimise neutral responses and facilitate clear judgements regarding content relevance.

Content validity was assessed using the Item Content Validity Index (I‐CVI) and the Scale Content Validity Index based on average agreement (S‐CVI/Ave). Consistent with established recommendations for expert panels comprising six or more members, an I‐CVI value of ≥ 0.78 was considered indicative of acceptable content validity. Consensus was further evaluated using median scores, interquartile ranges (IQR), and percentage agreement, with consensus predefined as a median score of ≥ 3.5, an IQR of ≤ 1.0, and agreement of ≥ 80%. An IQR of ≤ 1.0 indicated a high level of agreement by reflecting minimal variability in expert ratings across panel members. These criteria were applied concurrently to evaluate the relevance, clarity, and stability of expert judgements. Statements that did not satisfy one or more predefined validity or consensus criteria were conceptually revised based on expert feedback before being re‐evaluated in the subsequent Delphi round.

Descriptive statistics were used to summarise quantitative findings. Qualitative feedback provided by experts was analysed using directed content analysis. Comments were systematically coded, categorised according to the predefined model domains, and compared across experts to identify recurring suggestions regarding conceptual clarity, component definitions, interrelationships among model elements, and areas requiring conceptual refinement. Findings from the quantitative consensus analysis and qualitative content analysis were then integrated iteratively to guide refinement and establish the final validated conceptual model.

### 2.6. Ethical Considerations

This study received ethical approval from the Research Ethics Committee of Universitas Padjadjaran (approval no. 902/UN6.KEP/EC/2025). All participants received written information regarding the study objectives, procedures, and their rights as research participants prior to data collection. Written informed consent was obtained from all experts before participation. Participation was voluntary, and participants were informed of their right to withdraw from the study at any stage without consequence. To ensure confidentiality and anonymity, individual responses were deidentified during data analysis and reporting, and all study data were securely stored with access restricted to the research team.

## 3. Results

### 3.1. Expert Panel Characteristics

Eight experts participated in the Delphi study and completed both rounds, resulting in a retention rate of 100%. The panel represented diverse expertise across nursing leadership, caring science, nursing management, clinical leadership, and primary nursing practice. Participants included nursing academics, nurse managers, senior clinical nurses, and experienced primary nurses, providing complementary theoretical, managerial, and practice‐based perspectives for evaluation of the proposed model (Table 1).

### 3.2. Round 1: Content Validation of the Preliminary Model

The first Delphi round evaluated the relevance, clarity, and conceptual coherence of the preliminary self‐leadership‐based caring model. Twenty conceptual statements representing five model domains were assessed by the expert panel. Content validity analysis demonstrated strong agreement across all domains, with I‐CVI values ranging from 0.88 to 1.00 and an overall S‐CVI/Ave of 0.96, indicating excellent content validity. No statement was rejected or recommended for removal. Of the 20 conceptual statements evaluated, 12 were accepted without modification, whereas eight required minor conceptual refinement. The results of Delphi Round 1 are presented in Table 2.

**Table 2:** Content validity results in Delphi round 1.

Model domain	Mean I‐CVI	Key expert recommendations	Decision
Contextual enablers	0.94	Clarify the roles of ethical climate and interprofessional collaboration as facilitating conditions for self‐leadership enactment	Accepted with minor revisions
Core self‐leadership capacities	0.97	Strengthen the emphasis on professional reflection as a key self‐leadership capability	Accepted with minor revisions
Caring enactment	1.00	Reinforce caring as the central mechanism through which self‐leadership is translated into practice	Accepted
Accountable professional agency	0.94	Clarify scope‐bound autonomy, accountability, and ownership of care outcomes	Accepted with minor revisions
Practice outcomes	0.94	Further specify outcomes related to continuity of care, therapeutic relationships, quality of nursing care, and sustainable professional practice.	Accepted with minor revisions

*Note:* I‐CVI, Item Content Validity Index; S‐CVI/Ave, Scale Content Validity Index/Average.

Expert feedback consistently indicated that caring should be conceptualised not merely as a professional value but as the relational mechanism through which core self‐leadership capacities are translated into accountable professional practice. This conceptual refinement resulted in the repositioning of caring as caring enactment within the revised framework.

### 3.3. Conceptual Refinement Following Round 1

Qualitative feedback from Round 1 complemented the quantitative consensus findings by strengthening conceptual clarity and improving the coherence of relationships among model domains. Although experts supported the overall structure of the preliminary framework, they consistently highlighted the need to clarify the mechanism through which self‐leadership capacities are translated into professional nursing practice. Recommendations particularly emphasised the role of caring, the meaning of professional agency, the function of contextual enablers, and the contribution of reflexive feedback within the proposed model.

In response, expert feedback consistently indicated that caring should be conceptualised not merely as a professional value but as the central mechanism through which self‐leadership capacities are enacted in accountable professional practice. Consequently, professional agency was refined as accountable professional agency, characterised by responsibility, ownership of care, and scope‐bound autonomy. Contextual enablers were clarified as facilitating rather than determining conditions, while reflexive feedback was strengthened as a continuous learning and reinforcement process. These conceptual refinements integrated quantitative consensus with qualitative expert recommendations, resulting in a more coherent and process‐oriented framework that was subsequently redistributed for confirmation in Round 2.

### 3.4. Delphi Round 2: Consensus Confirmation

The revised model was redistributed to all experts for re‐evaluation in Round 2, and all panel members completed the assessment, resulting in a retention rate of 100%. Consensus was achieved across all model components and evaluation items, confirming that the conceptual refinements introduced after Round 1 were considered appropriate by the expert panel. Median scores ranged from 3.5 to 4.0, IQR ranged from 0.0 to 1.0, and agreement levels ranged from 87.5% to 100%, exceeding the predefined consensus criteria. The highest levels of agreement were observed for contextual enablers, core self‐leadership capacities, caring enactment, accountable professional agency, practice outcomes, reflexive feedback, and practical relevance to primary nursing. Although the integration of self‐leadership and caring theories received slightly lower agreement, all consensus indicators remained within acceptable thresholds, indicating that the theoretical integration was considered conceptually coherent and practically applicable. The stability of expert ratings and the absence of substantive recommendations for further modification confirmed that the revised framework had achieved conceptual stability and was accepted as the final conceptual model (Table 3).

**TABLE 3:** Delphi round 2 consensus results.

Model domain/evaluation item	Median	IQR	Agreement (%)	Consensus status
Contextual enablers	4.0	0.0	100.0	Achieved
Core self‐leadership capacities	4.0	0.0	100.0	Achieved
Caring enactment	4.0	0.0	100.0	Achieved
Accountable professional agency	4.0	0.0	100.0	Achieved
Practice outcomes	4.0	0.0	100.0	Achieved
Reflexive feedback	4.0	0.0	100.0	Achieved
Integration of Self‐Leadership and Caring Theories	3.5	1.0	87.5	Achieved
Practical Relevance to Primary Nursing	4.0	0.0	100.0	Achieved

*Note:* Consensus was predefined as median ≥ 3.5, IQR ≤ 1.0, and agreement ≥ 80%.

The Round 2 findings demonstrated that the revisions introduced following Round 1 successfully resolved the principal conceptual issues identified by the expert panel. The convergence of high quantitative consensus and the absence of additional substantive recommendations indicated that the revised framework had reached conceptual stability. Accordingly, the model was considered theoretically coherent, internally consistent, and sufficiently robust to serve as the final conceptual framework for self‐leadership based on caring in primary nursing practice.

### 3.5. Final Self‐Leadership Based on Caring Model

**FIGURE 1:**
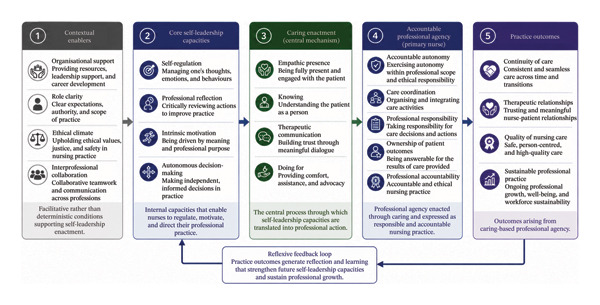
Final conceptual model of self‐leadership based on caring to strengthen primary nursing practice. Note. The model conceptualises caring enactment as the central mechanism through which core self‐leadership capacities are translated into accountable professional agency and practice outcomes. Practice outcomes subsequently reinforce future self‐leadership capacities through a reflexive feedback process that supports continuous professional development.

The final validated model represents the integration of quantitative expert consensus and qualitative conceptual refinement achieved through the two‐round Delphi process. It comprises five interconnected domains: contextual enablers, core self‐leadership capacities, caring enactment, accountable professional agency, and practice outcomes, supported by a reflexive feedback mechanism (Figure 1). The model conceptualises self‐leadership as a dynamic professional process in which caring enactment serves as the central mechanism translating self‐leadership capacities into accountable professional practice, supported by organisational and professional conditions that facilitate leadership development.

Within the model, caring enactment functions as the central relational mechanism through which core self‐leadership capacities are translated into accountable professional agency. This process is expressed through accountable autonomy, responsibility for care, clinical decision‐making, care coordination, and ownership of patient outcomes, which contribute to continuity of care, therapeutic relationships, quality of nursing care, and sustainable professional practice. Practice outcomes subsequently generate reflexive feedback that promotes reflective learning, reinforces future self‐leadership capacities, and supports continuous professional development.

## 4. Discussion

### 4.1. Understanding Self‐Leadership as a Caring‐Based Leadership Process

This study developed and conceptually validated a self‐leadership‐based caring model for primary nursing practice through a structured Delphi process. The validated framework suggests that self‐leadership should not be understood solely as an intrapersonal capability but as a professional process that becomes visible through everyday nursing practice [8, 9]. Across both Delphi rounds, experts consistently supported the integration of self‐leadership, caring, accountable professional agency, and practice outcomes within a coherent conceptual structure, indicating that leadership in primary nursing is enacted through clinical interactions, professional judgement, and responsibility for patient care rather than through formal authority alone [19, 34]. These findings further support the Delphi consensus that leadership emerges through the integration of self‐leadership capacities with caring processes rather than from individual competencies alone.

These findings align with contemporary nursing leadership scholarship, which increasingly recognises leadership as a relational, practice‐based, and context‐sensitive phenomenon embedded within frontline care delivery rather than restricted to managerial positions [3, 35, 36]. However, existing self‐leadership literature has largely focused on self‐regulation, motivation, and individual performance, providing limited explanation of how these capacities are translated into observable professional actions in clinical settings. The present model extends this perspective by proposing that leadership becomes visible when self‐leadership capacities are enacted through caring processes and expressed as accountable professional practice. In doing so, the model provides a nursing‐specific explanation of frontline leadership that integrates individual capability, relational practice, and professional responsibility within a single conceptual framework.

### 4.2. Caring as the Mechanism of Leadership Enactment

The most significant conceptual contribution of this study is the repositioning of caring from a professional value to a mechanism of leadership enactment. Watson and Swanson conceptualised caring as a fundamental ethical, relational, and disciplinary foundation of nursing practice, emphasising the centrality of human connection, responsiveness, and commitment to patient well‐being [10, 12]. Contemporary caring literature likewise emphasises caring as a central determinant of patient experiences, therapeutic relationships, and quality of care [37, 38]. Nevertheless, caring has predominantly been conceptualised as a professional value, moral commitment, or practice outcome rather than as a process through which leadership is enacted.

The Delphi findings suggest a different interpretation. This interpretation reflects the experts’ consensus that caring operationalises self‐leadership by transforming internal leadership capacities into observable professional actions within primary nursing practice. Experts consistently endorsed the view that self‐leadership capacities become professionally meaningful only when translated into action through caring processes. Within the validated model, caring functions as the pathway through which self‐regulation, professional reflection, intrinsic motivation, and autonomous decision‐making are transformed into observable nursing actions. Through empathic presence, knowing, therapeutic communication, and doing for, nurses enact leadership in ways that influence patient care, care coordination, and professional accountability [11, 13].

This interpretation extends both self‐leadership and caring theories. While conventional self‐leadership theory explains how individuals influence their own cognition, motivation, and behaviour, the present model explains how these internal capacities become visible in practice through caring relationships [31, 32]. Likewise, the model extends caring theory by positioning caring not only as a relational and ethical process but also as the mechanism through which leadership capacities are enacted in everyday nursing practice [10, 12]. Caring therefore functions as the critical link between internal leadership capacities and accountable professional action, providing a novel theoretical explanation of leadership within primary nursing.

### 4.3. Accountable Professional Agency as the Manifestation of Self‐Leadership Based on Caring

Another important contribution of the Delphi process was the refinement of professional agency into accountable professional agency. Throughout Round 1, experts consistently emphasised that agency should not be interpreted as unrestricted autonomy or individual authority. Instead, accountable professional agency was conceptualised as the capacity to exercise professional judgement, coordinate care, assume responsibility for decisions, and maintain accountability for patient outcomes within professional, ethical, and organisational boundaries [39, 40].

This distinction is particularly relevant in contemporary healthcare environments, where nurses are increasingly expected to demonstrate initiative, clinical judgement, and leadership while working within collaborative and highly regulated systems. Professional agency has been recognised as a critical component of nurses’ ability to influence care processes, advocate for patients, and respond effectively to complex clinical situations [41, 42]. However, the mechanisms through which such agency develops and becomes visible in practice have remained insufficiently explained. The validated model addresses this gap by proposing that accountable professional agency emerges through the enactment of self‐leadership based on caring. This finding explains why professional accountability should be understood as the outcome of caring enactment rather than merely an organisational expectation.

Within the model, caring enactment functions as the process through which self‐leadership capacities are translated into responsible professional action. Through caring relationships and practices, nurses enact leadership in the form of accountable autonomy, care coordination, professional responsibility, and ownership of patient outcomes [43, 44]. Consequently, leadership is not demonstrated merely by possessing self‐regulatory or motivational capacities but by translating those capacities into caring actions that directly influence patient care and professional practice [32]. By positioning accountable professional agency as the manifestation of self‐leadership based on caring, the model offers a nursing‐specific explanation of frontline leadership that reconciles autonomy with accountability and aligns leadership enactment with the core values of nursing practice.

### 4.4. Contextual Enablers as Facilitative Conditions for Leadership Enactment

The Delphi panel consistently highlighted the importance of contextual enablers, including organisational support, role clarity, ethical climate, and interprofessional collaboration. However, experts rejected deterministic interpretations suggesting that supportive environments alone generate leadership behaviours. Instead, the final model conceptualises contextual enablers as facilitative conditions that create opportunities for leadership enactment while preserving the active role of nurses as professional agents.

This interpretation is consistent with contemporary nursing management literature demonstrating that healthy work environments, supportive leadership structures, and positive organisational climates contribute to nurses’ engagement, empowerment, and leadership behaviours [17, 45]. Nevertheless, the findings suggest that favourable organisational conditions are necessary but not sufficient. Leadership emerges when nurses actively mobilise self‐leadership capacities and translate them into caring actions within clinical practice. In this sense, contextual enablers support, but do not determine, professional behaviour. Accordingly, nursing managers should strengthen leadership support, role clarity, ethical practice environments, interprofessional collaboration, and protected opportunities for reflective practice to optimise leadership enactment within primary nursing.

The distinction is particularly relevant to primary nursing practice, where nurses are expected to coordinate care, maintain continuity of care, and assume accountability for patient outcomes across the care trajectory [46, 47]. Such responsibilities require not only supportive organisational conditions but also the capacity to exercise professional judgement, reflection, and initiative. By conceptualising contextual factors as facilitative rather than deterministic, the model advances a balanced understanding of leadership as a situated process emerging from the interaction between organisational contexts, self‐leadership capacities, and caring enactment. This perspective suggests that efforts to strengthen frontline leadership should focus on both developing individual capabilities and fostering environments that enable leadership through caring practice.

### 4.5. Reflexive Feedback and Sustainable Leadership Development

A distinctive feature of the validated model is the inclusion of a reflexive feedback mechanism linking practice outcomes to future self‐leadership enactment. Throughout the Delphi process, experts consistently emphasised that leadership development should not be viewed as a linear progression but as an ongoing process of reflection, learning, and professional growth. Consequently, reflexive feedback was incorporated as a mechanism through which practice experiences continuously inform and strengthen future leadership capacities.

The model proposes that positive practice outcomes, including continuity of care, therapeutic relationships, quality of nursing care, and sustainable professional practice, create opportunities for reflection on professional actions and their consequences. Through this process, nurses evaluate their decisions, refine their clinical judgement, strengthen self‐regulatory capacities, and develop greater confidence in managing complex care situations [48, 49]. For example, reflecting on successful or challenging patient care experiences enables nurses to refine clinical judgement, strengthen self‐regulation, and improve future leadership behaviours. This interpretation is consistent with theories of reflective practice and experiential learning, which emphasise the role of experience as a catalyst for professional development and capability enhancement [50, 51].

The inclusion of reflexive feedback distinguishes the model from many conventional leadership frameworks that portray leadership as a predominantly linear process. Instead, the validated model conceptualises self‐leadership based on caring as a dynamic and cyclical phenomenon in which professional experiences continuously reinforce future enactment. This perspective is particularly relevant in contemporary healthcare environments characterised by rapid change, increasing complexity, and the need for continuous adaptation [52, 53]. By recognising reflection as a mechanism for sustaining leadership development, the model provides a theoretical explanation of how frontline nurses can progressively strengthen their leadership capacity through everyday caring practice.

### 4.6. Theoretical Contribution

This study advances nursing leadership scholarship by extending self‐leadership theory beyond its traditional intrapersonal focus and demonstrating how leadership capacities are translated into professional practice through caring enactment. These findings also demonstrate the value of the Delphi method for refining and validating conceptual nursing leadership models through systematic expert consensus. By integrating self‐leadership theory, caring theory, and primary nursing practice within a single explanatory framework, the model conceptualises leadership as a dynamic and cyclical process rather than a fixed individual attribute or formal organisational role.

### 4.7. Implications for Nursing Management

The validated model provides nursing managers with a practical framework for strengthening frontline leadership through the development of self‐leadership capacities and caring enactment. Its application may inform leadership development programmes, reflective supervision, mentorship initiatives, leadership coaching, and primary nursing implementation strategies. Managers may also use the model to strengthen organisational resources by promoting role clarity, supportive leadership, ethical practice environments, interprofessional collaboration, and protected opportunities for reflective practice. By strengthening both individual capabilities and organisational conditions, healthcare organisations may enhance accountable professional agency, continuity of care, quality of nursing care, and workforce sustainability.

### 4.8. Limitations

This study has several limitations. Although Delphi methodology prioritises expertise over sample size, the relatively small expert panel may have limited the diversity of perspectives represented during the validation process. In addition, all experts were recruited from a single national context, which may affect the transferability of the model to other healthcare systems and cultural settings. Furthermore, the study focused on conceptual development and validation rather than empirical testing of the proposed relationships.

### 4.9. Future Research

Future research should empirically evaluate the applicability of the model across diverse healthcare contexts and examine the relationships among contextual enablers, self‐leadership capacities, caring enactment, accountable professional agency, and practice outcomes. Future research should include implementation studies to evaluate the feasibility, adoption, and integration of the model into routine nursing practice. In addition, longitudinal and intervention studies are needed to determine the model’s effectiveness in strengthening leadership development, workforce sustainability, and quality of nursing care. Future studies should also examine the model across different cultural and healthcare systems to establish broader conceptual transferability.

## 5. Conclusion

This study developed and conceptually validated a self‐leadership based on caring model for primary nursing practice through expert consensus using the Delphi technique. The validated framework demonstrates that self‐leadership is not merely an individual capability but a professional process enacted through caring and supported by enabling organisational conditions. By positioning caring enactment as the central mechanism through which self‐leadership capacities are translated into accountable professional agency and practice outcomes, the model provides a nursing‐specific explanation of how frontline nurses operationalise leadership within everyday clinical practice.

The model further advances nursing leadership scholarship by conceptualising leadership as a dynamic and developmental process strengthened through reflexive feedback, which enables continuous professional learning and reinforces future self‐leadership capacities. As a theoretically grounded and expert‐validated framework, it provides a foundation for leadership development, primary nursing implementation, and future empirical research. Successful implementation of the model requires organisational support, including leadership support, role clarity, ethical practice environments, interprofessional collaboration, and opportunities for reflective practice, to strengthen self‐leadership, caring enactment, and accountable professional agency, thereby enhancing continuity of care, quality of nursing care, and workforce sustainability.

## Funding

The publication of this article was supported by Universitas Padjadjaran through the International Reputable Journal Publication Funding Program under Grant No. 1719/UN6.RKT/Kep/HK/2025.

## Conflicts of Interest

The authors declare no conflicts of interest.

## Data Availability

The data supporting the findings of this study are available from the corresponding author upon reasonable request.
